# Lateral prefrontal cortex is a hub for music production from structural rules to movements

**DOI:** 10.1093/cercor/bhab454

**Published:** 2021-12-30

**Authors:** Roberta Bianco, Giacomo Novembre, Hanna Ringer, Natalie Kohler, Peter E Keller, Arno Villringer, Daniela Sammler

**Affiliations:** UCL Ear Institute, University College London, London WC1X 8EE, UK; Otto Hahn Research Group Neural Bases of Intonation in Speech and Music, Max Planck Institute for Human Cognitive and Brain Sciences, Leipzig 04103, Germany; Neuroscience of Perception and Action Lab, Italian Institute of Technology (IIT), Rome 00161, Italy; Otto Hahn Research Group Neural Bases of Intonation in Speech and Music, Max Planck Institute for Human Cognitive and Brain Sciences, Leipzig 04103, Germany; Institute of Psychology, University of Leipzig, Leipzig 04109, Germany; Otto Hahn Research Group Neural Bases of Intonation in Speech and Music, Max Planck Institute for Human Cognitive and Brain Sciences, Leipzig 04103, Germany; Research Group Neurocognition of Music and Language, Max Planck Institute for Empirical Aesthetics, Frankfurt am Main 60322, Germany; Department of Clinical Medicine, Center for Music in the Brain, Aarhus University, Aarhus 8000, Denmark; The MARCS Institute for Brain, Behaviour and Development, Western Sydney University, Sydney, NSW 2751, Australia; Otto Hahn Research Group Neural Bases of Intonation in Speech and Music, Max Planck Institute for Human Cognitive and Brain Sciences, Leipzig 04103, Germany; Otto Hahn Research Group Neural Bases of Intonation in Speech and Music, Max Planck Institute for Human Cognitive and Brain Sciences, Leipzig 04103, Germany; Research Group Neurocognition of Music and Language, Max Planck Institute for Empirical Aesthetics, Frankfurt am Main 60322, Germany

**Keywords:** action hierarchy, inferior frontal gyrus, motor sequences, musical syntax, predictive coding

## Abstract

Complex sequential behaviors, such as speaking or playing music, entail flexible rule-based chaining of single acts. However, it remains unclear how the brain translates abstract structural rules into movements. We combined music production with multimodal neuroimaging to dissociate high-level structural and low-level motor planning. Pianists played novel musical chord sequences on a muted MR-compatible piano by imitating a model hand on screen. Chord sequences were manipulated in terms of musical harmony and context length to assess structural planning, and in terms of fingers used for playing to assess motor planning. A model of probabilistic sequence processing confirmed temporally extended dependencies between chords, as opposed to local dependencies between movements. Violations of structural plans activated the left inferior frontal and middle temporal gyrus, and the fractional anisotropy of the ventral pathway connecting these two regions positively predicted behavioral measures of structural planning. A bilateral frontoparietal network was instead activated by violations of motor plans. Both structural and motor networks converged in lateral prefrontal cortex, with anterior regions contributing to musical structure building, and posterior areas to movement planning. These results establish a promising approach to study sequence production at different levels of action representation.

## Introduction

Music, like speech, is a human sequential behaviour that is governed by combinatorial structural rules. These rules guide listeners' expectations during music perception (Pearce 2018; Koelsch et al. 2019), but also drive performers' movements during music production (Maidhof et al. 2009; Ruiz et al. 2011; Mathias et al. 2015; Bianco, Novembre, Keller, Kim, et al. 2016). One long-standing question is how the brain applies these abstract rules to motor behaviour (Lashley 1951). Here, by using a realtime imitation paradigm, we set out to identify the neural networks for abstract structural representations and their translation into movements during music production.

**Figure 1:**
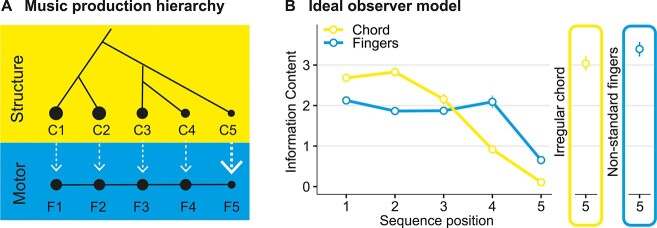
Model of hierarchical planning in music production. (*A*) Differentiating structural and motor levels of planning. Music-theoretical rules regulate the combination of structural units such as chords in musical sequences (C1–C5 in the yellow panel). Black lines illustrate local and temporally extended dependencies between chords. Successive dots of progressively smaller diameter indicate that the pool of possible chords that fit the preceding structural context reduces as the sequence unfolds. This gradually enhances the precision of structural planning, thereby facilitating performance. Motor plans of finger configurations (F1–F5 in the blue panel) are generated serially from one chord to another (black lines). Dashed white arrows illustrate the flexible key-to-finger mapping in piano performance. Successive dots of similar diameter across the sequence indicate that the evolving motor context of finger positions does not modulate motor plans beyond immediately adjacent acts. The choice of fingers can, however, be facilitated when structural plans are strong (e.g., at sequence end; bold white arrow). (*B*) Ideal observer model of structural and motor plans. For each chord (yellow) and finger configuration (blue) in a sequence, the Prediction by Partial Matching (PPM) model outputs the information content (IC) as a measure of the event unexpectedness given the preceding structural or motor context. The IC at the chord level gradually drops as the sequence unfolds suggesting a context effect on structure-level planning. Conversely, the IC at the motor level remains considerably stable throughout the sequence and only drops at the final chord when structural plans are precise. The yellow and blue boxes on the right show the sudden increase of IC when the structural fit of the final chord or the fingers used for playing this chord are, respectively, violated. Values are derived from standard sequences similar to those employed in the present study (see Materials and Methods).

Music is built on combinatorial structural rules, for example, the rules of harmony. They govern the arrangement of a limited set of musical elements (e.g., notes or chords) into virtually infinite varieties of musical sequences (Lerdahl and Jackendoff 1983; Swain 1995; Rohrmeier 2011). Similar to linguistic grammatical rules that define sentence structure, musical rules define which musical elements are likely to follow in a given context depending on local and temporally extended structural dependencies (Figure 1*A*) (Patel 2003; Pearce 2018; Koelsch et al. 2019). A wealth of research has established that listeners continuously apply these rules to the music they hear to form expectations about what notes or chords will come next (Tillmann 2012; Pearce 2018; Koelsch et al. 2019). In production, experienced performers similarly rely on these rules to anticipate future structural units in the music they play (for example, a C major chord at the end of a C major piece; Palmer and van de Sande 1993, 1995; Clarke 2001; Novembre and Keller 2011; Sammler, Novembre, et al. 2013). In previous work, we determined that structural planning during production of standard chord sequences heavily relies on the structural information of the global musical context: in an imitation task, musicians were faster and more accurate in playing structurally, i.e., harmonically, regular than irregular chords, more so when chords were embedded in long than short musical contexts (Novembre and Keller 2011; Sammler, Novembre, et al. 2013). This context-dependent facilitation indicates that structural plans become increasingly precise as the context unfolds, because the pool of harmonically likely next chords reduces with increasing structural information. But how is the structural plan of a sequence motorically implemented, movement by movement?

General models of action control posit that abstract representations of the global action structure (e.g., the steps needed to prepare coffee) incrementally activate single acts and actual movements (e.g., the hand configuration to open the coffee machine) at shorter time scales, namely at the time of their use (Lashley 1951; Schmidt 1975; Fuster 2001; Cooper and Shallice 2006; Rosenbaum et al. 2007; Grafton and Hamilton 2007; Koechlin and Summerfield 2007; Cisek and Kalaska 2010; Uithol et al. 2012; Diedrichsen and Kornysheva 2015; Hasson et al. 2015; Badre and Nee 2018; Burt et al. 2018). Similarly, playing music entails hierarchical levels of action planning that operate at different time scales and levels of abstraction (Figure 1*A*; Lashley 1951; Shaffer 1981; Todd 1985; Palmer 1989; Clarke 2001; Palmer and Pfordresher 2003). Superordinate abstract rules regulate the combination of structural units, such as chords, over extended timescales and sharpen context-based structural plans of which chord will come next in a sequence. Once the chord is planned, concrete motor parameters for its execution can be set, for example, the choice of finger configuration (note that the key-to-finger mapping is flexible in piano performance, that is, each key could be pressed with any finger). The selection of fingers often depends on local motor-anatomical considerations to optimize movement transitions from one chord to another (Clarke et al. 1997; Sloboda et al. 1998). Importantly, violations of such motor principles lead to increased execution times, that, as opposed to violations of structural plans, are not modulated by the length of the musical context (Bianco, Novembre, Keller, Scharf, et al. 2016; Bianco et al. 2018). This indicates that motor plans (i.e., the setting of motor parameters) are formed locally. At the same time, these local motor plans can be facilitated by higher-level structural plans (Todd 1985; Palmer 1989; Clarke 2001), particularly when structural plans are becoming increasingly precise towards the end of a sequence (Bianco, Novembre, Keller, Scharf, et al. 2016) (Figure 1*A*).

The different time scales of structural and motor planning can be formally described using computational approaches widely used to model expectations in auditory sequences (Pearce et al. 2010; Omigie et al. 2013; Barascud et al. 2016; Cheung et al. 2019; Gold et al. 2019; Bianco et al. 2020; Di Liberto et al. 2020; Quiroga-Martinez et al. 2020), under the assumption that perception and action, as sequential behaviours, rely on common processing principles (Hommel et al. 2001; Cooper 2019). A prominent computational model of sequence processing, based on the Prediction by Partial Matching (PPM) algorithm, learns the probabilistic composition of symbolic sequences through exposure to a training dataset. It then estimates the conditional probability (or degree of unexpectedness computed as information content - IC; negative log probability) of each event in new sequences based on multiple-order Markovian transition probabilities, i.e., based on a variable number of preceding events (Bunton 1996; Pearce 2005; Harrison et al. 2020). The probability with which chords or movements could be planned as a function of the structural or motor context can be estimated by training this model separately with the preceding chords or finger configurations (see Methods). Figure 1*B* shows that the planning of chords and finger configurations in standard 5- chord sequences depends on temporally extended vs. local transition probabilities, respectively: the IC of consecutive chords gradually decreases with growing structural context (yellow line), whilst for fingers it remains considerably stable throughout the sequence (blue line). The drop in IC for the fingers only at the end of the sequence - when structural plans are highly precise - may reflect the top-down effect of structural on motor planning. Structural or motor violations at the last position, i.e., a harmonically irregular chord or non-standard finger configuration, lead to sudden increases of IC (yellow and blue frames in Figure 1*B*). The brain responses associated with one or the other of these violations should reflect re-planning at high structural or lower motor levels of action representation.

Neural models of lateral prefrontal cortex (LPFC) function may provide clues as to which brain areas are involved in such multilevel planning processes. Growing evidence indicates a hierarchical organization of action control along the anterior–posterior axis of LPFC, suggesting that progressively more anterior LPFC regions control actions at increasingly abstract levels and over longer temporal scales (Botvinick 2007; Koechlin and Summerfield 2007; Badre 2008; Badre and D’Esposito 2009). For example, while single movements are represented in primary motor cortex (Yokoi et al. 2018), representations of movement sequences are found in premotor cortex (PMC) and inferior/middle frontal gyrus (IFG/MFG) (Koechlin and Jubault 2006; Yokoi and Diedrichsen 2019), with more abstract rules extending further into more anterior portions of LPFC (Koechlin and Jubault 2006; Badre et al. 2010). Moreover, connectivity profiles of anterior and posterior LPFC regions differ, suggesting their involvement in functionally distinct large-scale neural networks (Passingham et al. 2002; Anwander et al. 2007; Clos et al. 2013; Neubert et al. 2014; Hartwigsen et al. 2019). Notably, an important role of the IFG in integrating abstract information over time is highlighted by music perception studies (for review, see Asano et al. 2021), which consistently showed the sensitivity of this area to long-distant structural dependencies between musical elements during listening (Koelsch et al. 2002, 2005; Tillmann et al. 2006; Bianco, Novembre, Keller, Kim, et al. 2016; Cheung et al. 2018). This suggests that the IFG is a plausible area to support abstract structural processes also during music production (Fitch and Martins 2014; Bianco, Novembre, Keller, Kim, et al. 2016).

**Figure 2:**
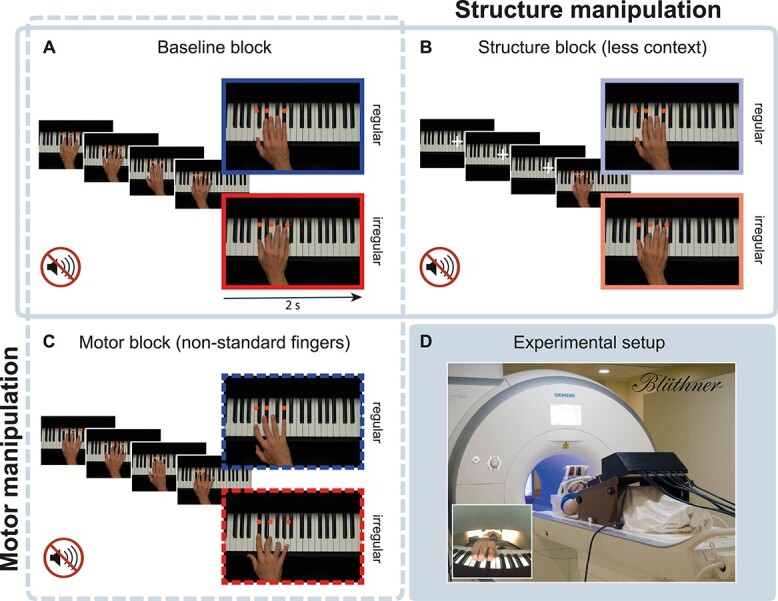
Experimental design and setting. (*A*–*C*) Pianists executed novel chord sequences as shown in series of photos on screen. The last chord of all sequences (enlarged photos) was manipulated in its structural regularity (regular/irregular). (*A*, *B*) To address the influence of structural rules on action planning, structurally regular and irregular chords were embedded in long (*A*, “baseline block”) or short contexts (*B*, “structure block”). (*A*, *C*) To address low-level motor planning, structurally regular and irregular chords had to be performed with standard (*A*, “baseline block”) or nonstandard finger configurations (*C*, “motor block”). Effects of structural and motor planning were assessed in separate models (solid and dashed frames, respectively). (*D*) Pianists executed these chord sequences on an MR-compatible piano in a 3 T scanner by imitating the hand in the photos both in terms of the keys pressed and the fingers used for playing (i.e., also structural and finger errors had to be performed as displayed on screen). No sound was played to avoid confounding brain activity associated with the auditory processing of music. The pianist’s hand was filmed with a fish lens camera (inset on the bottom left).

Here, we combine models of music cognition and action control to identify and compare the neural networks for abstract structural planning versus actual motor plans during a music imitation task (Novembre and Keller 2011; Bianco, Novembre, Keller, Scharf, et al. 2016; Bianco et al. 2018). We acquired behavioral, functional, and diffusion-weighted neuroimaging data from expert pianists instructed to perform novel musical chord sequences on an MR-compatible piano without sound (Fig. 2*D*). Performance was guided by series of photos of a pianist’s hand (Fig. 2*A*–*C*). Sequences were constructed so as to violate 1) the structural plans of chords presented at the end of long and short musical contexts and 2) the motor plans of which fingers to use for the execution of these chords. Importantly, the mapping between chords and fingers was not fixed in the stimuli, allowing for the dissociation of structural and motor level processes: 50 different chords were arranged into different sequences, and different finger configurations mapped onto the reoccurrences of a given chord (see Materials and Methods). While fingering violations activated a frontoparietal network, structural violations activated a frontotemporal network. Notably, LPFC was part of both networks with anterior regions contributing to high-level structural plans and posterior regions to low-level motor plans. LPFC may thus constitute the hub at the interface between cognitive and motor networks where abstract structural rules are converted into movements.

## Materials and Methods

### Participants

We present data from 26 pianists (8 female; mean age = 25.6 years, *SD* = 4.1). Sample size was chosen based on our previous fMRI study involving a similar paradigm and number of stimuli (Bianco, Novembre, Keller, Kim, et al. 2016). Participants had a minimum of 6 years of piano training in classical Western tonal music (range = 6–28 years, mean = 17.1 years, *SD* = 5.5) and had started to play the piano at an average age of 8.1 years (*SD* = 3.8, range = 2–17 years). Data were acquired from 11 more pianists that were however excluded from the analysis because they were not able to perform the task (*N* = 3 had less than 50% accuracy) or because of technical issues during fMRI data acquisition (*N* = 8). Written informed consent was obtained from each participant prior to the study that was approved by the local ethics committee of the University of Leipzig (016-15-26 012 015).

### Paradigm

We used an established paradigm to identify brain areas associated with structural and motor planning during piano performance (Novembre and Keller 2011; Bianco, Novembre, Keller, Scharf, et al. 2016; Bianco et al. 2018). Pianists played unrehearsed chord sequences on an MR-compatible piano by imitating, with their right-hand, actions of a model hand shown in series of photos (Fig. 2). No sound was played to avoid confounding brain activity associated with the auditory processing of music.

Structure-level planning was identified by manipulating the structural regularity of sequence-final chords (regular/irregular) and the length of the musical context (long/short context) in a 2 × 2 design (see solid frame and “structure manipulation” in Fig. 2*A*,*B*). More precisely, structural plans were manipulated in strength and were violated in half of the trials by placing harmonically irregular chords (right bottom photos in Fig. 2*A*,*B*) at the end of long or short sequences (Fig. 2*A*,*B*, respectively). The use of different sequence lengths capitalizes on the dependency of structural planning on sequence context (Sammler, Novembre et al. 2013; Bianco, Novembre, Keller, Scharf, et al. 2016 and Fig. 1*B*): A long context induces stronger structural plans on the identity of the final chord than short sequences. Brain areas associated with structural planning should show stronger activity changes for irregular chords at the end of long, as opposed to short, sequences.

Motor planning and its interaction with structural plans were investigated by manipulating the choice of fingers (standard/nonstandard fingers) and the structural regularity of final chords (regular/irregular) in a 2 × 2 design (see dashed frame and “motor manipulation” in Fig. 2*A*,*C*). Motor planning was disrupted by introducing unusual finger configurations for playing the final chords (Fig. 2*C*). Brain areas that support motor planning based on the preceding movement and regardless of the structural regularity should show overall stronger activity for final chords played with nonstandard versus standard fingers. Brain areas in which structural plans facilitate lower-level motor plans should show stronger activity for finger violations when chords were structurally regular compared with when they were irregular.

### Stimuli

Stimuli consisted of chord sequences that were presented as photos of a hand playing chords on a piano (i.e., one photo for each chord; Fig. 2) (Bianco, Novembre, Keller, Scharf, et al. 2016; Bianco et al. 2018). Each chord was presented with the same duration of 2 s. The interval between sequences was jittered between 3 and 9 s (mean = 5.6 s) during which a black screen was displayed. A total of 26 different 5-chord sequences formed the basic stimulus pool, composed according to the rules of classical harmony in six different tonalities (D, E, Bb, Ab, A, and Eb major). Each chord consisted of three notes to be played with the right hand. The mapping between chords and fingers was not fixed: For example, the chord eb-ab-c was played with 3 different finger configurations (1-2-4/1-3-5/1-2-3); also, 6 different finger configurations were used for a total of 50 different chords arranged into 26 sequences. This is different from numerous studies on motor sequence production with fixed mappings, that is, one specific key for each finger (e.g., Yokoi and Diedrichsen 2019; de Manzano et al. 2020). Note that in such sequences, structural and motor plans (i.e., which keys to press and which fingers to use) cannot be dissociated.

The 26 basic sequences were manipulated in terms of structural regularity and finger configuration of the final chord and in terms of sequence length (five or two chords) to obtain six conditions. As it is illustrated in Figure 2, the event preceding the last chord (i.e., the penultimate chord) was identical in all six conditions and cannot account for activity differences. Conditions were presented in blocks as follows: First, “baseline” blocks (Fig. 2*A*) contained the 26 five-chord sequences ending with either a harmonically regular (a Tonic chord) or irregular chord (a Neapolitan chord, namely ﻿a minor subdominant with a diminished sixth instead of a fifth, rarely used in classical harmony to resolve a musical sequence). Final chords were controlled for visual appearance as in Bianco, Novembre, Keller, Scharf et al. (2016) by balancing the average amount of black and white keys across conditions. Furthermore, chords that appeared as Tonic in one sequence also appeared as Neapolitan in another sequence. Second, to identify brain regions involved in abstract structural planning, all 5-chord sequences of the “baseline” blocks (long context) were truncated and only the last two chords of each sequence (short context) were presented in “structure” blocks (Fig. 2*B* and solid frame “structure manipulation”). In these blocks, the two photos constituting the short sequences were preceded by three photos of a piano with no hand but a white fixation cross on it, during which pianists were asked to perform a thumb opposition task with the right hand (as in Haslinger et al. 2005). That is, before playing the last two chords, pianists had to touch their thumb with index, middle, ring, and little finger (in that order and back for the duration of the fixation cross). This task, involving coordination between fingers in time and space similarly to playing chords but without musical associations, was included to minimize general differences in sensorimotor activity between long and short sequences, inevitably characterized by a different number of movements before the final target chord. Finally, to identify brain regions involved in low-level motor planning, the final chords of the long sequences used in the “baseline” block had to be played with nonstandard finger configurations in “motor” blocks (Fig. 2*C* and dashed frame “motor manipulation”). The finger patterns used as motor violations (2-3-5 and 2-4-5) do occur in real piano performance (see, as an example, Opus 28 Nr 9 by Chopin), but they are less frequent in simple chord progressions as those used in the present study, as confirmed by expert pianists’ ratings (see Bianco, Novembre, Keller, Scharf, et al. 2016).

### Modeling Sequences of Chords versus Sequences of Movements

An unsupervised statistical learning model (Harrison et al. 2020) based on the Prediction by Partial Matching (PPM) algorithm (Cleary and Witten 1984) was used to describe the probability of chord harmonic functions and fingers associated with each event in our sequences. This was done to formally support previous experimental data showing a context-effect on structural rather than motor planning (Bianco, Novembre, Keller, Scharf, et al. 2016; Bianco et al. 2018) and to motivate our contrasts of interest in the fMRI data analysis (see below). Input to the model is symbolic in nature. Therefore, stimuli were translated in two sets of symbols: One set denoted the chord functions defined as the structural relationship of a chord to the tonal center of the sequence (e.g., Tonic and Dominant) and its inversion (determined by which of the three notes composing the chord was the lowest). The second set of symbols pertained to the finger configurations. Sixty standard 5-chord progressions were used to train the model in two separate runs to learn the statistics associated either with the chord functions or with the finger configurations. This training set was composed analogously to the sequences used in the experiment (both in terms of structure and fingers) but included tonalities not used in the present study (C, G, B, F, Db, Gb major). Only structurally and motorically correct sequences were used for training. The training set’s statistics were then applied to the sequences used in the present study to estimate the information content (IC) of each chord based on the structural context, and of each finger configuration based on motor context. Moreover, we also estimated IC in sequences containing chord or finger violations, to show the expected sudden increases in IC. Results are shown in Fig. 1*B*.

### Procedure

Pianists were instructed to watch and simultaneously imitate the chord sequences played by the hand in the photos: They were instructed to reproduce both, keys pressed and fingers used. This means, they also had to reproduce the structural irregularities or use nonstandard fingers, exactly as displayed on screen. A mirror mounted on the head coil allowed them to see the photos projected onto a screen at the head-end of the MR-scanner. For execution, they used a custom-built MR-compatible piano with 27 weighted keys manufactured by Julius Blüthner Pianofortefabrik GmbH (Leipzig, Germany; Fig. 2*D*). Weighted keys increase the ecological validity of the performance by giving the users a similar touch experience as playing on a real piano. The experiment was run in the absence of musical sound, that is, participants played the chord sequences without receiving auditory feedback of their motor actions (and likewise, no sounds were associated with the photos). Key presses, velocity, and key releases were sensed optically using a light-emitting diode, a matching phototransistor, a pair of fiber optic cables, and a reflector for each key of the MR-piano as in Hollinger et al. (2007). All electronic components of the piano were located in the room adjacent to the scanning room, with the optical cables entering the scanning room through the wall (wave-guide). The piano was positioned on a slightly tilted wooden stand over the participant lying supine in the bore of the MR-scanner. Pianists’ finger movements were monitored and recorded through an MR-compatible camera with fisheye lens (12 M camera, MRC Systems) placed on top of the piano. This allowed offline analysis of finger errors committed by pianists.

The 26 stimuli were repeated in all their manipulation variants across 6 miniblocks. All blocks (each ~8 min) consisted of 26 trials and were organized as follows: Two blocks of the type “baseline” contained long sequences with structurally regular/irregular final chords played with standard fingers (Fig. 2*A*); two blocks of the type “structure” contained short sequences with structurally regular/irregular endings played with standard fingers (Fig. 2*B*); two blocks of the type “motor” contained structurally regular/irregular chords at the end of long sequences but played with nonstandard fingers (Fig. 2*C*). The order of blocks was fully randomized across participants. Trials within each block were presented in pseudorandom order with the constraint that no more than three sequences of the same condition followed each other. Stimulus presentation was controlled with presentation software (version 14.9, Neurobehavioral Systems, Inc.). Pianists’ key presses on the MR-piano were recorded by custom-written Python software running on a Linux computer.

To acquaint participants with the task in the scanner, a mock training session was run about one week before the scanning day. During this presession, participants were trained with a different set of sequences in different tonalities (G, B, F, Db) in a mock scanner on a MIDI-keyboard (M-Audio Keystation 49e, inMusic GmbH).

### MR Data Acquisition

The experiment was carried out in a 3.0-Tesla Siemens PRISMA whole-body magnetic resonance scanner (Siemens AG) using a 32-radiofrequency-channel head coil. *Functional magnetic resonance images* were acquired using a *T*_2_^*^-weighted 2D echo planar imaging (EPI) sequence with TE = 30 ms and TR = 2000 ms. About 240 volumes were acquired for each block, with a square FOV of 210 mm, with 37 interleaved slices of 3.2-mm thickness and 15% gap (3 × 3 × 3.68 mm^3^ voxel size) aligned to the AC-PC plane, and a flip angle of 77°.

High-resolution *T_1_*-weighted anatomical images and diffusion-weighted images of the participants were either taken from the database of the Max Planck Institute or acquired in the context of the fMRI experiment. Diffusion-weighted MR data were available for 21 pianists. Anatomical images were recorded using a 3D MP2RAGE sequence (TI_1_ = 700 ms, TI_2_ = 2500 ms, TE = 2.03 ms, TR = 5000 ms) with a matrix size of 240 × 256 × 176, with 1-mm isotropic voxel size, flip angle_1_ of 4°, flip angle_2_ of 8°, and GRAPPA acceleration factor of 3. Diffusion-weighted data were acquired with a twice-refocused spin echo EPI sequence (TE = 100 ms, TR = 12 900 ms, 88 axial slices without gap, FOV = 220 mm, matrix size = 128 × 128, iPAT = 2) with 1.71875-mm isotropic voxel size. Diffusion-weighting was isotropically distributed along 60 diffusion-encoding gradient directions with a *b*-value of 1000 s/mm^2^. Additionally, seven images without diffusion-weighting (b0) were recorded evenly distributed across scan time and served as anatomical reference for offline motion correction.

### Behavioral Data Analysis

Performance of the last chord was analyzed as in previous studies using this paradigm (Novembre and Keller 2011; Sammler, Novembre, et al. 2013; Bianco, Novembre, Keller, Scharf, et al. 2016). Trials were included in the analysis when 1) the penultimate and final chord of the sequence were imitated correctly, both in terms of keys and fingers, 2) when the three keys in the penultimate and in the final chord were pressed synchronously (i.e., no more than 150-ms elapsed between the first and the last of the 3 keystrokes), and 3) when response times (RTs) of the final chord were below 3000 ms. RTs were the averages of the three keystrokes of the final chord time-locked to the onset of the last photo in the sequence. Fingers used by the participants were analyzed through off-line inspection of the video recordings of their hands. For each participant, RTs that deviated by more than 2 *SD*s from the mean across conditions were discarded from the analysis. Based on these exclusion criteria, an average of 69 ± *SD* 15.5% of the total number of trials remained to be analyzed across participant. RTs and number of errors (key and finger errors) were used as dependent variables. Key and finger errors were assumed to reflect distinct cognitive processes associated with the structural and the motor planning, respectively (as in Bianco, Novembre, Keller, Scharf, et al. 2016). RT data of one participant were lost during data acquisition, while errors could be reconstructed through inspection of the video (showing near perfect performance).

To address the rule-based structural planning, we ran two-way analyses of variance (ANOVA) with the repeated-measures factors STRUCTURE (regular/irregular chords) and CONTEXT (long/short) on RTs and number of key errors including the trials from the “baseline” and “structure” blocks. To address low-level motor planning and its interaction with higher-level structural plans, we ran a two-way ANOVA with the repeated-measures factors STRUCTURE (regular/irregular chords) and MOVEMENT (standard/nonstandard fingers) on RTs including the trials from the “baseline” and “motor” blocks. Number of errors was analyzed with an analogous ANOVA, but with the additional within-subject factor ERROR TYPE (key/finger errors). ANOVAs were implemented in the R environment (version 0.99.320) using the “ezANOVA” function (Lawrence MA 2016). Post hoc *t*-tests were used to resolve significant interactions, and Bonferroni-correction was applied based on the number of comparisons.

### fMRI Data Analysis

fMRI data were analyzed with SPM12 (Welcome Trust Centre for Neuroimaging, University College, London, UK, http://www.fil.ion.ucl.ac.uk/spm/software/spm12) using standard spatial preprocessing procedures. These consisted of slice time correction (using cubic spline interpolation), spatial realignment, coregistration of functional and anatomical data (uniform tissue-contrast image masked with the second inversion image from the MP2RAGE sequence), spatial normalization into the Montreal Neurological Institute (MNI) stereotactic space that included resampling to 2 × 2 × 2 mm voxel size. Finally, data were spatially low-pass filtered using a 3D Gaussian kernel with full-width at half-maximum (FWHM) of 8 mm and temporally high-pass filtered with a cut-off of 1/128 Hz to eliminate low-frequency drifts.

The evoked hemodynamic response to the onset of the final chord was modeled for each of the six conditions (the regular/irregular chords in the “baseline,” “structure,” and “motor” blocks) as boxcars convolved with a hemodynamic response function (HRF). All trials were included in the brain data analysis to maximize statistical power. Error trials and estimated motion realignment parameters were added to this design as covariates of no interest to regress out residual motion artifacts and to increase statistical sensitivity. To control for motor effort due to the transition to structural and movement violations, RTs were used as a duration-modulated parametric regressor orthogonalized to the stimulus onset regressors (following Grinband et al. 2008).

Whole-brain random-effects models were implemented to account for within-subject variance. Statistical parametric maps for each of the six conditions (one-sample *t*-tests against implicit baseline) were generated for each participant in the context of the general linear model (GLM) for use in the second-level group analysis.

We then ran two models with 2 × 2 within-subject full factorial designs to identify brain regions associated with the different levels of the action hierarchy. The first model contained the trials from the “baseline” and “structure” blocks and the factors STRUCTURE (regular/irregular chord) and CONTEXT (long/short). The interaction of STRUCTURE × CONTEXT should unveil brain areas modulated by the strength of the structure plan. The second model with the factors STRUCTURE and MOVEMENT included the trials from the “baseline” and “motor” blocks. The main effect of MOVEMENT (standard > nonstandard fingers) should identify brain regions involved in low-level motor planning based on the preceding movement regardless of musical structure, and an interaction of STRUCTURE × MOVEMENT should reveal brain areas where high-level structural plans facilitate lower-level motor plans.

For statistical thresholding, we adopted a widely used nonparametric estimation of statistical threshold that addresses emerging concerns of balancing whole-volume type I and type II errors (Slotnick et al. 2003; Slotnick 2017; Lohmann et al. 2018; Eklund et al. 2019; Noble et al. 2020). A Monte Carlo simulation run in MATLAB (1000 iterations, no volume mask) suggested a cluster extent threshold of ≥46 resampled voxels at a voxel-level uncorrected *P*-value of 0.001 to yield a threshold corrected for multiple comparisons of *P* < 0.05 (Slotnick et al. 2003; code available at https://drive.google.com/file/d/16HVUD-PZaEpwHoZE99YXDxhcuLawjW7O/view?usp=sharing). Anatomical labeling was based on the SPM anatomy toolbox (Eickhoff et al. 2005).

### Diffusion Data Analysis

To specify connectivity patterns of LPFC for structural and motor levels of action control, we used frontal along with temporal and parietal activation peaks of the two fMRI analyses as seed and target regions in probabilistic tractography and estimated the most likely underlying white matter pathways. Temporal target regions were selected for their involvement in musical structure processing (MTG; Koelsch et al. 2002; Tillmann et al. 2006; Sammler, Koelsch, et al. 2013), while parietal target areas were chosen for their involvement in music production (SPL; Bianco et al., 2016) and general motor control (Andersen and Buneo 2002; O’Reilly et al. 2013). Processing of diffusion data and anatomical reference images was done in FSL (version 5.0.9, FMRIB, University of Oxford, www.fsl.fmrib.ox.ac.uk/fsl), SPM12, and LIPSIA (Max Planck Institute for Human Cognitive and Brain Sciences; Lohmann et al. 2010). Diffusion-weighted images were first motion-corrected using rigid-body transformations based on the seven (b0) nondiffusion-weighted reference images and then registered to the *T*_1_-weighted anatomical images resampled to diffusion space with 1.72 × 1.72 × 1.72 mm resolution. Subsequently, fiber orientation was estimated in each voxel by means of the software module BEDPOSTX (with standard options) in FSL using a crossing fiber model with up to two directions per voxel (Behrens et al. 2007).

Seed regions for tractography were obtained by first projecting MNI group coordinates in left IFG [−44, 24, −4] and MTG [−58, −20, −8], bilateral PrCG [−56, 6, 26; 58, 10, 20] and SPL [−36, −36, 52; 34, −42, 58] into each participants’ diffusion MRI space. Coordinates that fell into sulci or gray matter were shifted to the nearest white matter voxel defined by fractional anisotropy (FA) values of ≥0.3. Spheres with 5-mm radius around the selected coordinate served as seed regions. To distinguish dorsal and ventral pathways, coronal slices crossing dorsal tracts at y = 3 to 5 and y = −2 to 0 and crossing ventral tracts at y = 3–5 and y = −22 to −19 were manually marked as waypoint masks in MNI space and then morphed into participants’ native space.

Probabilistic tractography between IFG-MTG and PrCG-SPL via dorsal or ventral waypoint masks in each hemisphere was computed bidirectionally using the PROBTRACKX2 module in FSL, with 5000 streamlines per seed region voxel, a curvature threshold of 0.2, step length of 0.5, and maximum number of steps of 2000. Resulting tractography images were cleaned for random connections (threshold at 5% of the image’s maximum intensity value), normalized to MNI space, binarized, and summed. Group-level images were slightly smoothed (Gaussian filter with 0.5-mm FWHM) and corrected for filter-induced blurring at the rim (binarization threshold at 0.0001). Plots of pathways found in more than 50% of the participants were generated using brainGL (http://braingl.googlecode.com). Fiber tracts were labeled following the JHU White-Matter-Tractography Atlas in FSL (Hua et al. 2008).

Finally, to estimate the functional relevance of the identified pathways, we set up two multiple regression models using fractional anisotropy (FA) of the relevant tracts as predictors for behavioral performance changes following structural or motor violations, respectively. Therefore, we averaged FA values of all voxels with FA > 0 within a fiber tract per participant. Voxels that were part of more than one fiber tract (e.g., of both AF/SLF and IFOF), or that were not reliably part of the given fiber tract in at least 50% of participants, were discarded. As dependent variable, we calculated a so-called SP-index (structural–priming index) for each participant, reflecting the behavioral STRUCTURE × CONTEXT interaction. More precisely, mean RTs in the four conditions in “baseline” and “structure” blocks were entered into the following formula: SP = (RT_irregular-long_ − RT_regular-long_) − (RT_irregular-short_ − RT_regular-short_). A higher SP-index indicates stronger structural planning based on the available (long) context information. Moreover, we calculated the overall slowing of RTs during nonstandard fingering by subtracting mean RTs in the “baseline” from those in “motor” blocks for each participant, reflecting the behavioral main effect of MOVEMENT.

## Results

### Structural Planning

**Figure 3:**
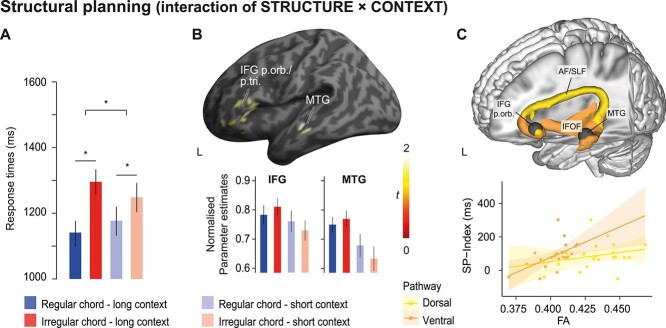
Structural planning. (*A*) Behavioral performance. Mean RTs associated with structurally regular and irregular chords in the long and short contexts (“baseline” vs. “structure” blocks). Error bars indicate ±1 SEM. ^*^indicates significant effects *(P* < 0.05). (*B*) fMRI data. Full factorial analysis of trials from the “baseline” and “structure” blocks with the factors STRUCTURE (regular/irregular) and CONTEXT (long/short). Execution of structurally irregular chords evoked stronger activity than regular chords when embedded in the long compared with the short context, as revealed by the interaction of STRUCTURE × CONTEXT. Activated regions included left inferior frontal gyrus (IFG) and MTG; min-max normalized contrast estimates at 90% CI from local maxima are depicted below the brain plots for left IFG (p. orb./BA 47 [−44 24 −4] and left MTG (BA21) [−58 −20 −8]. Error bars indicate ±1 SEM. Threshold for display: *P*_voxel_ < 0.001; cluster extent ≥46 resampled voxels corresponding to *P*_cluster_ < 0.05 according to Slotnick et al. (2003). p. tri.: pars triangularis; p. orb.: pars orbitalis; SFG: superior frontal gyrus. (*C*) Probabilistic tractography. Group overlay of dorsal (yellow) and ventral fiber tracts (orange) connecting anterior IFG and posterior MTG. Only voxels with fibers in more than 50% of the participants are depicted. Seed regions for probabilistic tractography are colored in gray. Visualization of the fiber tracts was done in brainGL (http://braingl.googlecode.com). The bottom panel shows that the FA values of the ventral pathway positively correlated with the strength of the behavioral STRUCTURE × CONTEXT interaction (SP-Index), reflecting stronger structural planning based on the available (long) context information.

Behavioral data from “baseline” and “structure” blocks are shown in Figure 3*A*. Structurally irregular chords were performed overall more slowly than regular chords [main effect of STRUCTURE: *F*(1,24) = 22.05, *P* < 0.001, np^2^ = 0.48], but more so in the long than in the short context [interaction of STRUCTURE × CONTEXT: *F*(1,24) = 34.11, *P* < 0.001, np^2^ = 0.59]; no main effect of CONTEXT [*F*(1,24) = 0.14, *P* = 0.713, np^2^ < 0.01; irregular vs. regular in long context: *t*(24) = 6.20, *P* < 0.001; irregular vs. regular in short context: *t*(24) = 2.62, *P* = 0.003]. This suggests that structural planning was more precise in long than short sequences causing higher costs—longer reaction times—when the plan had to be revised in case of a structural violation of the last chord. In terms of accuracy, more key errors were committed in structurally irregular than regular chords [main effect of STRUCTURE: *F*(1,24) = 17.98, *P* < 0.001, np^2^ = 0.43; mean number of errors ± *SD*: regular chord = 0.38 ± 2.75; irregular chord = 1.20 ± 1.19], but we found no STRUCTURE × CONTEXT interaction [*F*(1,24) = 1.32, *P* = 0.262, np^2^ = 0.05].

Figure 3*B* shows the brain results of the full factorial analysis of the “baseline” and “structure” blocks with the factors STRUCTURE (regular/irregular) and CONTEXT (long/short). Based on our previous work, the behavioral data, and the model predictions in Figure 1*B*, we focused on the interaction of STRUCTURE × CONTEXT, that is, greater activity differences between regular and irregular chords in the long than in the short context. The interaction involved the left anterior IFG (pars triangularis and orbitalis, BA45/47) and middle temporal gyrus (MTG, BA 21) (Table 1). The activity pattern in these clusters consistently showed higher activity for irregular compared with regular chords when embedded in a long context (compare red and blue bars in the parameter estimates in Fig. 3*B*). For completeness, the main effects of CONTEXT and STRUCTURE are shown in Supplementary Figure 1*A* and Supplementary Table 1.

**Table 1:** Structural planning (interaction of structure and context)

Gyrus or region	Hem	BA	*k*	*x*	*y*	*z*	*Z*-value
**Frontal inf. (pars orbitalis)**	**L**	**47**	**434**	**−44**	**24**	**−4**	**4.01**
“		47		−48	44	−8	3.87
Frontal inf. (pars triangularis)		45		−40	30	10	3.60
**Temporal mid.**	**L**	**21**	**110**	**−58**	**−20**	**−8**	**3.94**
“		21		−48	−20	−8	3.58
**Caudate**	**R**	**—**	**47**	**12**	**8**	**10**	**3.82**

Whole-brain activation cluster sizes (*k*), MNI coordinates (*x*, *y*, *z*), and Z-scores for the Structure × Context contrast (Structurally irregular > regular chords in the long context—Structurally irregular > regular chords in the short context) (*P*_voxel_ < 0.001; correction for multiple comparisons to *P* < 0.05 was obtained using a voxel cluster extent threshold procedure that led to minimum cluster extent threshold of 46 resampled voxels). BA: Brodmann area, Hem.: hemisphere, inf.: Inferior, mid.: Middle.

Probabilistic fiber tractography with seeds in left IFG and MTG showed that these regions are structurally interconnected primarily via the inferior fronto-occipital fascicle (IFOF; see Fig. 3*C*), while the arcuate/superior longitudinal fascicle (AF/SLF III) did not consistently reach into anterior IFG across participants. The multiple regression analysis showed that mean FA of the left IFOF (standardized β = 0.624, *t*(20) = 2.581, *P* = 0.019), but not the left AF/SLF (standardized *β* = −0.111, *t*(20) = −0.461, *P* = 0.650), significantly predicted the SP-index: The higher FA in left IFOF, the stronger was the behavioral STRUCTURE × CONTEXT interaction in RTs, that is, the stronger were participants’ context-based structural predictions (full model adj. *R*^2^ = 0.244, *F*(2,18) = 4.22, *P* = 0.031).

### Motor Planning

**Figure 4:**
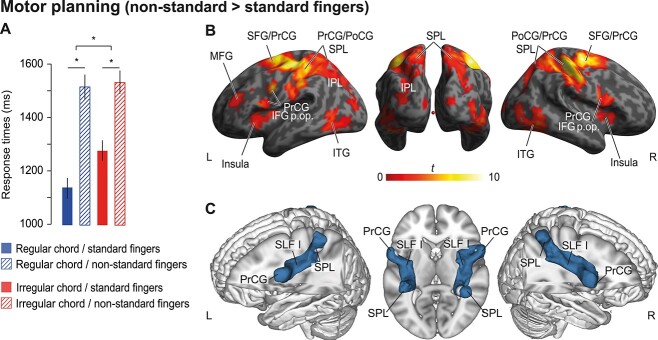
Motor planning. (*A*) Behavioral performance. Mean RTs associated with motorically standard (filled bars) or nonstandard fingers (striped bars) of structurally regular (blue) and irregular chords (red) (“baseline” vs. “motor” blocks). Error bars indicate ±1 SEM. ^*^indicates significant effects (*P* < 0.05). (*B*) fMRI data. Full factorial analysis of the “baseline” and “motor” blocks with the factors STRUCTURE and MOVEMENT. Execution of chords with nonstandard compared with standard movements elicited stronger activity in bilateral frontoparietal action areas. Threshold for display: *P*_voxel_ < 0.001; cluster extent ≥46 resampled voxels corresponding to *P*_cluster_ < 0.05 according to Slotnick et al. (2003). p. op.: pars opercularis; PrCG: precentral gyrus; PoCG: postcentral gyrus; SFG: superior frontal gyrus; SPL: superior parietal lobule; IPL: inferior parietal lobule; ITG: inferior temporal gyrus. (*C*) Probabilistic tractography. Group overlay of dorsal fiber tracts (blue) connecting PrCG and SPL. Only voxels with fibers in more than 50% of the participants are depicted. Seed regions for probabilistic tractography are colored in light gray. Visualization of the fiber tracts was done in brainGL (http://braingl.googlecode.com).

Figure 4*A* shows participants’ RTs in “baseline” and “motor” blocks. Nonstandard finger patterns were imitated more slowly than standard finger patterns [main effect of MOVEMENT: *F*(1,24) = 177.60, *P* < 0.001, np^2^ = 0.88]. Likewise, RTs were longer for structurally irregular than regular chords [main effect of STRUCTURE: *F*(1,24) = 24.33, *P* < 0.001, np^2^ = 0.50]. Moreover, we found a significant interaction of STRUCTURE × MOVEMENT [*F*(1,24) = 30.34, *P* < 0.001, np^2^ = 0.56]: Nonstandard (compared with standard) finger patterns slowed down performance, more so when chords were structurally regular [*t*(24) = 12.92, *P* < 0.001] compared with when they were irregular [*t*(24) = 10.93, *P* < 0.001]. This suggests that regular structure may have primed standard motor plans more than in the irregular structure condition, so that motor plans were costlier to be revised in case of nonstandard fingers. In terms of accuracy, a 2 × 2 × 2 repeated-measures ANOVA with the factors MOVEMENT, STRUCTURE, and ERROR TYPE (key/finger errors) showed that overall more errors were committed when either of the planning levels was violated [main effect of MOVEMENT: *F*(1,24) = 22.09, *P* < 0.001, np^2^ = 0.48; main effect of STRUCTURE: *F*(1,24) = 17.25, *P* < 0.001, np^2^ = 0.42]. In particular, the interaction of MOVEMENT × ERROR TYPE [*F*(1,24) = 11.70, *P* = 0.002, np^2^ = 0.33] indicates that violations of motor plans were associated with a greater increase of finger than key errors [nonstandard vs. standard finger condition, finger errors: *t*(24) = 4.46, *P* < 0.001; key errors: *t*(24) = 2.46, *P* = 0.042], while violations of structural plans tended to induce a greater increase of key than finger errors [STRUCTURE × ERROR TYPE [*F*(1,24) = 3.68, *P* = 0.067, np^2^ = 0.13]; irregular vs. regular chord condition, key errors: *t*(24) = 4.16, *P* < 0.001; finger errors: *t*(24) = 2.22, *P* = 0.07]. Neither a MOVEMENT × STRUCTURE interaction nor a three-way interaction with ERROR TYPE was found.

Figure 4*B* shows the brain results of the full factorial analysis of the “baseline” and “motor” blocks with the factors STRUCTURE and MOVEMENT. Based on our previous work, the behavioral data, and the model predictions in Figure 1*B*, we focused on the main effect of MOVEMENT and its interaction with STRUCTURE. For completeness, the main effect of STRUCTURE is shown in Supplementary Figure 1*B* and Supplementary Table 1. We found a main effect of MOVEMENT in a broadly distributed set of frontoparietal regions. These included bilateral IFG (pars opercularis, BA44) and precentral cortices (PrCG, BA6), insula, SFG, and left MFG (BA46), as well as bilateral postcentral gyrus (PoCG), superior parietal lobule (SPL), left inferior parietal lobule (IPL), bilateral inferior temporal gyrus (ITG), and lobules VI, VIIb, and VIII of the right cerebellum (see Table 2 and Fig. 4*B*). The interaction of STRUCTURE × MOVEMENT showed one cluster in right sensorimotor regions (BA 3: [38, −36, 66]). In line with the behavioral data (Fig. 4*A*), the activity pattern in this cluster showed a greater effect of finger violation (nonstandard vs. standard finger patterns) when chords were structurally regular compared with when they were irregular (Supplementary Fig. 1*B*).

**Table 2:** Motor planning (nonstandard > standard fingers)

Gyrus or region	Hem	BA	*k*	*x*	*y*	*z*	*Z*-value
**Precentral**	**R**	**6**	**27 113**	**38**	**−14**	**64**	**Inf**
Postcentral		3		38	−32	52	Inf
Supplementary motor area		6		4	−2	54	Inf
Parietal sup.		2/40		34	−42	58	6.97
Fusiform		37		32	−44	−20	5.93
Temporal inf.		37		50	−60	−10	5.39
Cerebellum lobule VI		−		28	−50	−28	5.59
Precentral	L	6		−38	−10	62	7.80
Postcentral		3		−38	−24	52	Inf
Supplementary motor area		6		−6	−2	52	Inf
Parietal sup.		40		−36	−36	52	7.06
Fusiform		37		−38	−44	−18	5.75
Cerebellum lobule VI		−		−26	−50	−26	6.30
**Precentral/frontal inf. (pars opercularis)**	**L**	**6/44**	**2304**	**−56**	**6**	**26**	**7.24**
Insula (anterior)		48		−36	14	6	5.56
“		48		−40	0	10	5.05
**Precentral**	**R**	**6/44**	**1575**	**58**	**10**	**20**	**5.77**
Insula (anterior)		48		40	0	14	5.40
“		48		36	2	6	4.52
**Cerebellum lobule VIII**	**R**	**—**	**66**	**16**	**−68**	**−50**	**5.00**
Cerebellum lobule VIIb		**—**		34	−72	−50	3.60
“		**—**		10	−74	−44	3.25
**Occipital pole**	**R**	**17**	**111**	**14**	**−98**	**12**	**3.80**
**Lingual gyrus**	**L**	**17**	**76**	**−2**	**−82**	**−8**	**3.75**
**Occipital pole**	**L**	**17**	**93**	**−4**	**−100**	**12**	**3.68**
“		17		−6	−102	2	3.39
**Frontal mid.**	**L**	**45**	**692**	**−46**	**38**	**28**	**5.11**
Frontal inf. (pars triangularis)		45		−40	32	8	3.55

Whole-brain activation cluster sizes (*k*), MNI coordinates (*x*, *y*, *z*), and Z-scores for the Nonstandard > Standard Movement contrast (*P*_voxel_ < 0.001; correction for multiple comparisons to *P* < 0.05 was obtained using a voxel cluster extent threshold procedure that led to minimum cluster extent threshold of 46 resampled voxels). BA: Brodmann area, Hem.: hemisphere, inf.: Inferior, mid.: Middle, sup.: Superior.

Fiber tractography with seeds in bilateral PrCG and SPL showed dorsal connections via the SLF I (see Fig. 4*C*). No ventral connection was found. Mean FA of neither left nor right SLF predicted the RT changes following motor violations (full model adj. *R*^2^ = −0.091, *F*(2,18) = 0.16, *P* = 0.849; left SLF: standardized *β* = −0.085, *t*(20) = −0.172, *P* = 0.865; right SLF: standardized *β* = −0.053, *t*(20) = −0.107, *P* = 0.916).

### Comparison of Structural versus Motor Levels of Action Planning

**Figure 5:**
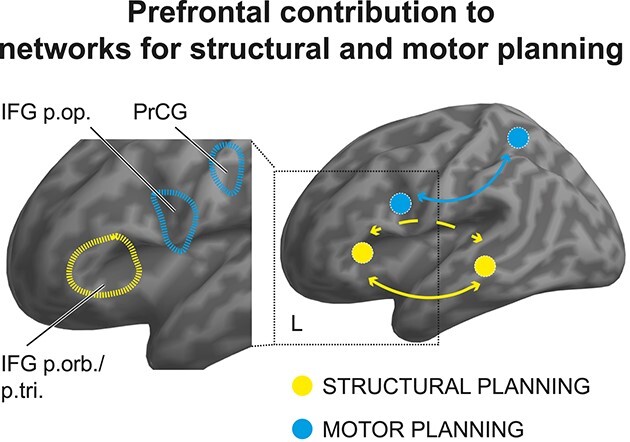
Dual networks and prefrontal contribution to structural and motor planning during music production. Anterior frontal and posterior temporal regions ventrally connected via the IFOF (less consistently via the AF/SLF; dashed) support rule-based structure processing during production of novel musical sequences (yellow). Conversely, posterior frontal and parietal regions dorsally connected via SLF I support the planning and execution of motor movements. The inset on the left suggests an anterior-to-posterior “gradient” along the left LPFC from abstract structural planning in pars triangularis and orbitalis of IFG (BA45/BA47) to actual motor planning in PrCG (BA6) and pars opercularis of IFG (BA44). p. op.: pars opercularis; p. tri.: pars triangularis; p. orb.: pars orbitalis; PrCG: precentral gyrus; BA: Brodmann area.

Figure 5 (right panel) summarizes our findings showing the frontotemporal network for structural planning (yellow) and the frontoparietal network for motor planning (blue) of musical actions. The two networks hardly overlap. Zooming in on frontal regions (left panel) further illustrates more fine-grained differences along the anterior-to-posterior axis of LPFC: rule-based structural planning was best captured by activity in anterior IFG (pars triangularis and orbitalis, BA 45/BA47), while motor planning evoked bilateral activity in posterior IFG (pars opercularis, BA 44) and premotor cortices (PrCG, BA 6).

## Discussion

We identified the neural networks of abstract structural and concrete motor planning in pianists during the imitation of novel musical chord sequences on a muted MR-compatible piano. We found two networks involved in one or the other level of action planning: Left anterior IFG (BA45/47) and posterior MTG (BA21) were activated by structure violations in long more than in short sequences and were interconnected ventrally via IFOF. Bilateral posterior IFG/PrCG (BA44/6) and parietal areas were activated by motor violations and were interconnected dorsally via SLF. In line with models of hierarchical action control, the combined data demonstrate the multilevel contribution of anatomically distinct cognitive and motor networks to the production of complex actions. Importantly, LPFC is identified as a hub where both networks converge, and where abstract structural representations may be transformed into sequential motor behavior.

### Rule-Based Structural Planning of Musical Sequences Involves a Frontotemporal Network

Execution was slower for structurally irregular than regular chords more so in long than short sequences (Fig. 3*A*), indicating performance facilitation when plans were met, but increased costs when plans were violated and had to be revised (Novembre and Keller 2011; Sammler, Novembre, et al. 2013; Bianco, Novembre, Keller, Scharf, et al. 2016). Notably, it is in line with the predictions of the computational model (Fig. 1*B*) that this effect was stronger in longer sequences, indicating that temporally extended structural dependencies govern the planning of chord sequences with increasing precision as the context unfolds.

Similar to the behavioral effects, structural violations activated the left anterior IFG (BA45/47) and left MTG (BA21) more strongly in long than in short sequences (Fig. 3*B*), indicating a role of these areas in enduring abstract representations of the structure governing the sequence at hand. This interpretation aligns with the predictions of the computational model (Fig. 1). It also fits with recent findings on the involvement of the LPFC (with stronger left hemispheric contribution) and the temporal lobe in maintaining temporal and contextual information for the execution of structured action sequences such as coffee making (Shahnazian et al. 2021). The left-lateralization of the present network further aligns with functional accounts that associate the left IFG with the general cognitive control of memory (Badre and Wagner 2007). According to these views, this area is broadly involved in abstract mechanisms of retrieval and selection of goal-relevant representations to guide and constrain actions through stored knowledge. The activity in the MTG may mark the location where structural knowledge is retrieved to support the pianists’ actions. This area has been indeed linked with high musical expertise and stronger representations of musical structure in perception studies (Zatorre et al. 1998; Janata et al. 2002; Haslinger et al. 2005; Seung et al. 2005; Bianco, Novembre, Keller, Kim, et al. 2016).

The exact format of the structural information that is processed in the left IFG and MTG remains to be determined. It is possible that pianists relied on motor and/or auditory imagery of the expected behavioral outcome to build structural plans (for review, Zatorre and Halpern 2005; Zatorre et al. 2007; Novembre and Keller 2014). Our results do not yield evidence that structural plans were derived within the motor system itself, and musicians were able to plan structural chords without auditory feedback (Repp 1999; Finney and Palmer 2003), opening the possibility of a rather “amodal” schematic representation of structural plans. The localization of effects in MTG rather than auditory core or belt areas adds weight to this possibility. However, it cannot be excluded that structural plans were guided by anticipations of the auditory outcome (Mathias et al. 2017) associated with the schematic content (Jebb and Pfordresher 2016) in line with previous studies showing left MTG activity during silent piano performance (Zhang et al. 2017; Martin et al. 2018).

Overall, our findings suggest potentially similar mechanisms underlying predictions based on long-distance structural dependencies in perception and production (Cooper 2019). In perception studies, frontotemporal areas have been consistently associated with the processing of structural violations, and they are thought to integrate past and current information to generate predictions about forthcoming events (Zatorre et al. 1998; Maess et al. 2001; Koelsch et al. 2002, 2005; Tillmann et al. 2003, 2006; Seung et al. 2005; Vuust et al. 2011; Kim and Sikos 2011; Koelsch 2011; Sammler et al. 2011; Zatorre and Salimpoor 2013; Musso et al. 2015; Bianco, Novembre, Keller, Kim, et al. 2016; Di Liberto et al. 2020). Although these perception studies often report a bilateral contribution, lesion studies have shown the need of intact left frontotemporal regions to process structural violations during listening (Sammler et al. 2011). Here, we show for the first time a similar network during music production. However, our production network extended slightly more anteriorly into pars orbitalis of IFG (BA47) and more ventrally into MTG, compared with activation peaks reported with auditory paradigms. These often fall into pars opercularis and triangularis (BA44/45) of IFG (Asano et al. 2021) and superior temporal gyrus (STG) (Koelsch 2005). We consider it unlikely that the present anterior focus in LPFC and ventral shift in the temporal lobe is a matter of modality (perception vs. production). Rather we attribute it to the tighter control of the structural violations for low-level (sensory and motor) features in the present than in previous fMRI studies. Our comparison of structural effects between long and short sequences may have better dissociated the abstract analysis of long-range structural dependencies from (spurious) local sensorimotor processes.

Finally, the diffusion data argue for the IFOF (Fig. 3*C*) as the most likely network connection between IFG and MTG. The IFOF directly connected both regions and showed a correlation with the strength of the structural plans formed by the musicians based on the context. Connections via the AF/SLF were less consistent across participants and no brain–behavior correlation was found. Only little is known about the relevance of white matter pathways between frontotemporal regions in music-structural processing, and evidence comes mostly from studies on acquired or congenital amusia. In music perception, both ventral (IFOF) and dorsal (AF/SLF) pathways have been associated with the ability to process musical structure (Musso et al. 2015), which is impaired after damage to these fiber tracts in acquired and congenital amusia (AF/SLF Loui et al. 2009; Chen et al. 2015, 2018; Peretz 2016; Sihvonen et al. 2017; IFOF: Sihvonen et al. 2017; Wang et al. 2017). In music production, available tractography studies explored the role of AF/SLF but have focused mostly on audiomotor coupling (Halwani et al. 2011; Engel et al. 2014) rather than music-structural processing. Hence, the present data are the first to highlight a left-lateralized ventral pathway linking IFG and MTG as most likely anatomical scaffold to support knowledge-driven, rule-based computations not only in perception but also in production of novel musical sequences.

### Motor Planning Involves a Frontoparietal Network

Performance was overall slower when chords had to be executed with nonstandard fingers (Fig. 4*A*), reflecting higher costs on motor planning imposed by violations of local motor principles. The brain data mirrored these results in a frontoparietal network including posterior IFG and PrCG (BA44/6), primary motor areas, the SPL/IPL, and lobules VI, VIIb, and VIII of the right cerebellum (Fig. 4*B*, Table 2), indicating the role of these areas in processing local motor transitions. The inclusion of RTs as a duration-modulated parametric regressor in the brain data analysis should exclude the interpretation that these effects may merely reflect motor effort. Instead, frontoparietal areas have been associated with the integration of sensorimotor information, prediction of motor outcomes and motor preparation, both for simple actions (Grèzes and Decety 2001; Williams et al. 2007; Papitto et al. 2020) as well as sequential finger movements (Yokoi et al. 2018; Yokoi and Diedrichsen 2019). Likewise, the most dorsal branch of the SLF that we found to interconnect these regions (Fig. 4*C*) has been associated with hand motor control and movement selection (Schulz et al. 2015), although our study did not find correlations with behavior. Finally, the observed cerebellar areas have been consistently associated with sensorimotor tasks, the planning and execution of single and sequential finger movements (for reviews, see Stoodley and Schmahmann 2009; Buckner 2013; King et al. 2019), including the production of music (e.g., Sergent et al. 1992; Lotze et al. 2003; Segado et al. 2018; Kita et al. 2021).

Beyond this fronto-parieto-cerebellar network, we also found activations in SFG (overlapping with the presupplementary motor area, pre-SMA) evoked by nonstandard fingers (in Supplementary Figure 1 and Supplementary Table 2). This pattern may reflect general error detection and response inhibition processes when the brain detects deviations from intended behaviors by estimating the mismatch between predicted and actual outcomes (Obeso et al. 2013; Wessel and Aron 2017; Gabitov et al. 2020). The additional recruitment of the anterior insula may indicate participants’ greater awareness of this salient error type and conscious response adaptation, with a possible impact on the arousal or affective state of the performers (Ullsperger et al. 2010).

Interestingly, both the behavioral and the neural results further revealed an interaction between movement and musical structure (Fig. 4*A*). This interaction suggests that, in addition to serial motor-anatomical principles (Clarke et al. 1997; Sloboda et al. 1998), also higher structural levels can prime low-level motor plans, in line with the predictions of the computational model (bold white arrow in Fig. 1*A*, and sudden drop of IC in the blue line in Fig. 1*B*). Motor violations slowed down both the execution of structurally regular and irregular chords, indicating that local motor principles were violated. However, this effect was stronger when chords were structurally regular, reflecting the priming of low-level motor parameters by strong high-level structural plans at the end of the sequence. The brain data mirrored this interaction in the right sensorimotor cortex (BA3; Supplementary Fig. 1*B*). The contribution of BA3 may reflect the role of this area in the somatosensory anticipation of the structurally primed movement. The ipsilateral contribution of this area may stem from a saturation of processes in the contralateral area leading to the additional recruitment of its ipsilateral homolog. This is in line with previous observations of ipsilateral versus contralateral somatosensory regions being more sensitive to anticipatory modulation effects during perceptual tasks (Van Ede et al. 2014). Altogether, the data argue for a hierarchical organization of action plans (Lashley, 1951), in which abstract structural representations can top-down facilitate movements.

On a methodological note, this cross-talk between levels may explain why our previous functional connectivity work (Bianco, Novembre, Keller, Kim, et al. 2016; corresponding to the main effect of structure shown in Supplementary Figure 1 and Supplementary Table 2) found the dorsal frontoparietal network for structurally irregular chords even though this study used only standard fingers: The highly precise structural plans may have resulted in violations of the just preactivated motor plans in frontoparietal areas. This highlights the importance of comparing the effect of structural violations across long and short sequences, as done in our current paradigm, to dissociate abstract structural from motor processes.

### Anterior and Posterior Frontal Contribution to Structural Rules and Movements

We found two distinct anterior and posterior subregions of the LPFC associated with representations of high-level musical structure and low-level elementary movements, respectively. This is in accordance with the functional anterior-to-posterior LPFC gradients postulated by models of action control (Miller and Cohen 2001; Koechlin et al. 2003; Wood and Grafman 2003; Badre and Nee 2018; Rouault and Koechlin 2018), based on neuroimaging studies revealing progressively more anterior activity in LPFC during response to progressively more complex stimuli (reviewed by Koechlin & Summerfield, 2007). For example, while movement sequences in response to simple sensory cues evoked activity in motor and premotor regions (BA4/6) (Koechlin et al. 2003; Koechlin and Jubault 2006; Badre and D’Esposito 2007), more complex sequences coordinated across nested temporal frames (Nee and D’Esposito 2017; Shahnazian et al. 2021) or embedded in hierarchical patterns (Koechlin and Jubault 2006) recruited anterior frontal regions (BA44/45/9 up to BA46/10). Our findings further suggest that the selection of single acts or movements in the posterior IFG/PrCG may be coordinated by higher-level internal representations of the abstract sequence structure held in the anterior IFG. We thus speculate that IFG may constitute the hub where conceptual and motor networks converge, and abstract structural representations are transformed into sequential motor behavior. Tracking the temporal and causal dynamics of this transformation along this putative gradient and over the course of learning is an interesting prospect for future research.

### Parallels to Speech Production

The idea that IFG is a key region at the interface between cognitive and motor networks parallels recent models of speech production (Garagnani and Pulvermüller 2013; Long et al. 2016; Flinker and Knight 2018; Hage 2018; Matchin and Hickok 2020). These models suggest that the conversion of abstract linguistic structures, such as sentences or words, into chains of articulatory movements relies on the interaction between the MTG as a syntactic hub (Meyer and Friederici 2016), and anterior frontal regions (in particular pars triangularis of IFG) that interface with premotor/motor areas where speech is motorically implemented. These models are supported by studies showing linguistic structure processing in anterior frontal regions (syntactic sentence structure in BA45: Segaert et al. 2013; Giglio et al. 2021; word generation and syllabification in BA44: Indefrey and Levelt 2004; Bourguignon 2014; Flinker et al. 2015) and brain activity that propagates from these regions to more posterior, precentral areas where lower-level articulatory movements are represented (Edwardsa et al. 2010; Uddén and Bahlmann 2012; Flinker et al. 2015).

Overall, these parallels between spoken language and music production support the idea that the left IFG plays a domain-general role in sequential behaviors (Fitch and Martins 2014; Bornkessel-Schlesewsky et al. 2015; Rouault and Koechlin 2018) by acting as a multimodal association zone or cortical hub (Friederici and Singer 2015) that links cognitive and sensorimotor networks. Our approach using production of musical sequences is promising to further illuminate this link. First, as opposed to speech entailing mouth movements, piano playing minimizes movement artifacts during neural signal recording, and it also allows the expected auditory outcome of an action to be easily manipulated/suppressed. Second, because musical sequences are composed of discrete elements, high-level structural and low-level motor processing can be easily controlled, parametrized, and computationally modeled. This will allow research to move from paradigms where production is primed to studies with free generation and improvisation.

## Conclusion

Understanding how actions are neurally represented at different hierarchical levels is a first crucial step to understand what enables humans to flexibly generate the variety of action sequences we use every day to communicate and interact with the world. While exciting theoretical and experimental advances have been achieved in understanding the generative power of human-specific, rule-based cognitive abilities (Wilson et al. 2017), it remains an open question why we ultimately generate one action sequence, and not another, among the infinite range of sequences we are able to conceive. Our work with musical sequences grounds the anatomical bases of production of rule-based actions within a dual network architecture. Further studies are needed to detail the timing of the recruitment of these networks, and how the constituent brain areas interact with one another during music production. Research in this direction combined with modeling work can increase our understanding of how complex sequential behaviors like speech and music gain the flexibility needed for meeting the demands of real-life interaction.

## Supplementary Material

FigureS1_accepted_211109_bhab454

TableS1_accepted_211109_bhab454

TableS2_accepted_211109_bhab454

## Data Availability

The datasets and scripts for this study can be found in the OSF repository (https://osf.io/b5cav/?view_only=ad78b46b1b4144d58afb3e3e4791d8f3).

## Notes

We thank the pianists who participated in this experiment and to Bruno Pace who lent a hand in stimulus preparation. We also thank Christopher J. Steele, Avrum Hollinger, and Julius Blüthner Pianofortefabrik GmbH for constructing the piano and Sven Gutekunst and Jöran Lepsien for technical support. *Conflict of Interest*: On behalf of all authors, the corresponding author declares no financial and nonfinancial competing interests.
