# Supplementary material for: Lateral prefrontal cortex is a hub for music production from structural rules to movements
Source: Cereb Cortex. 2021 Dec 30;32(18):3878–95. doi: 10.1093/cercor/bhab454 (PMC9476625; doi:10.1093/cercor/bhab454)
Supplement: FigureS1_accepted_211109_bhab454 [file figures1_accepted_211109_bhab454.docx]

**Supplementary Material**

**
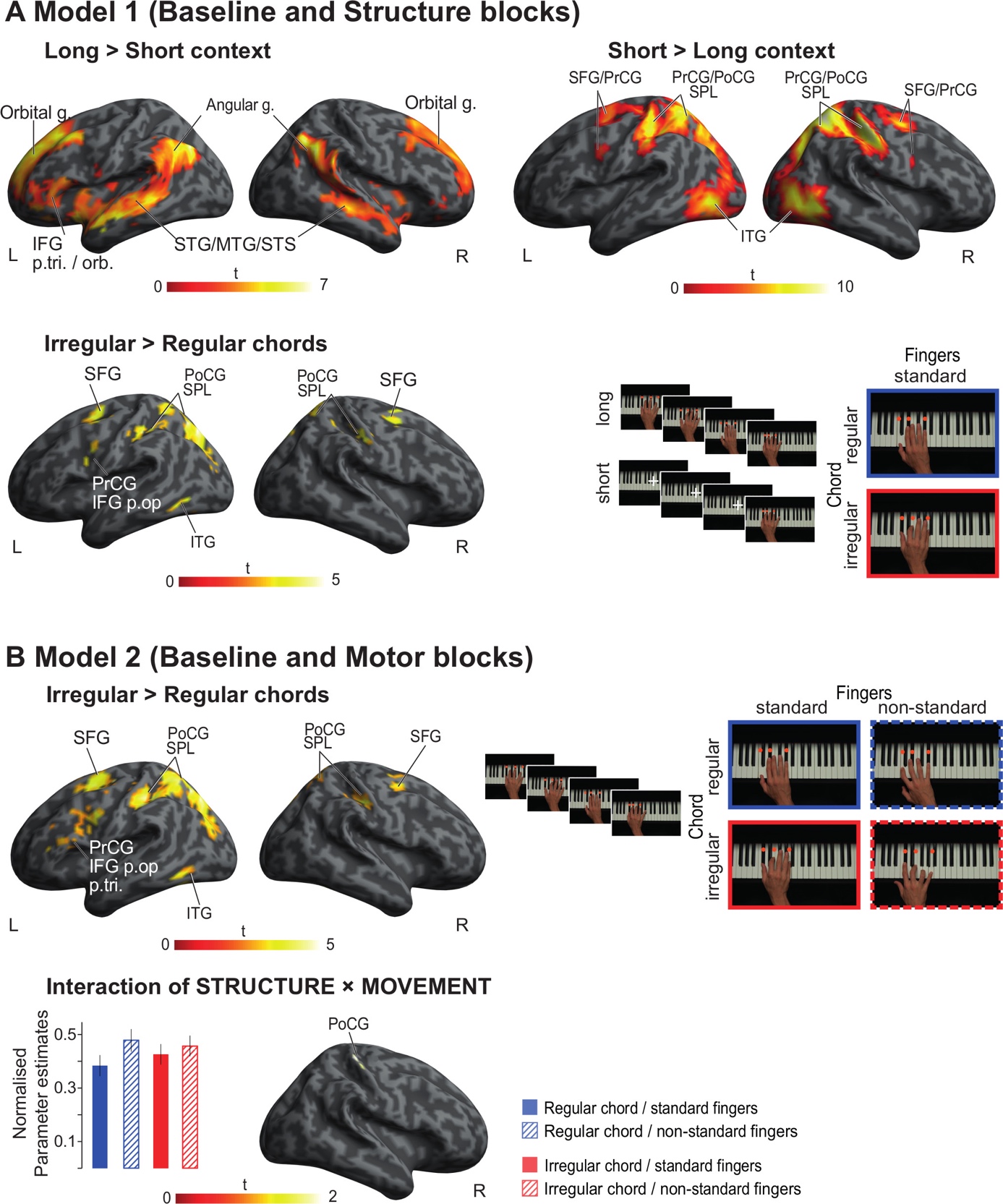
**

***Figure S1. (A) Full factorial analysis of trials from the ‘baseline’ and ‘structure’ blocks (Model 1). Long > Short context:*** *long sequences evoked stronger activity in frontal, superior temporal, and inferior parietal areas than short sequences. Left IFG and bilateral temporal activity may reflect stronger structural representations in long sequences, along with stronger auditory imagery reported previously in musicians performing or observing performances without sound* (Haslinger et al. 2005; Bangert et al. 2006)*.* ***Short > Long context:*** *short sequences evoked stronger activity in premotor, parietal, inferior temporal, and visual-occipital areas than long sequences. This activity pattern might reflect the predominance of motor over structural planning along with stronger reliance on visual cues when structural information was limited. Note, however, that both these contrasts should be interpreted with caution because long and short sequences – although identical in terms of penultimate and final chords – differed in their initial phase (imitation of three chords vs. thumb opposition task while seeing a white fixation cross).* ***Irregular > Regular chords:*** *irregular chords evoked stronger left frontal and posterior parietal activity than regular chords.* *Note that anterior frontal activity associated with structural planning (the IFG pars triangularis, BA45) is plausibly weakened by the presence of short sequences in which structural expectations were low.* ***(B) Full factorial analysis of trials from the ‘baseline’ and ‘motor’ blocks (Model 2). Irregular > regular chords:*** *similar to Model 1, irregular chords evoked greater activity than regular chords in bilateral fronto-parietal action areas, regardless of the fingers used to play the chords. Specifically, the areas included left precentral gyrus (PrCG, BA6) and inferior frontal gyrus (IFG, pars opercularis and triangularis, BA44/45), bilateral superior frontal gyrus (SFG), postcentral gyrus (PoCG), superior parietal lobule (SPL) and inferior temporal gyrus (ITG). The posterior parietal activity mirrors effects found in the main effect of MOVEMENT and may indeed reflect the violation of motor plans triggered by structural plans.* ***Interaction of STRUCTURE × MOVEMENT****: the right PoCG (BA3) showed a greater effect of finger violations (non-standard vs. standard finger patterns) when chords were structurally regular compared to when they were irregular. This may reflect the somatosensory anticipation of the structurally primed movement, and is in line with hierarchical organisation of action plans (Lashley, 1951), in which structural plans can top-down facilitate motor plans (see main text for more details). BA: Brodmann area.*
