# Supplementary material for: Lateral prefrontal cortex is a hub for music production from structural rules to movements
Source: Cereb Cortex. 2021 Dec 30;32(18):3878–95. doi: 10.1093/cercor/bhab454 (PMC9476625; doi:10.1093/cercor/bhab454)
Supplement: TableS1_accepted_211109_bhab454 [file tables1_accepted_211109_bhab454.docx]

| **Table S1. Full factorial analysis of trials from the ‘baseline’ and ‘structure’ blocks (Model 1).** | | | | | | | |
| --- | --- | --- | --- | --- | --- | --- | --- |
| Gyrus or region | Hem | BA | k | x | y | z | Z-value |
| ***Long > Short context*** |  |  |  |  |  |  |  |
| **Angular Gyr.** | **L** | **39** | **36234** | **-56** | **-68** | **32** | **7.25** |
| Temporal Sup. |  | 21/22 |  | -50 | -2 | -18 | 5.79 |
| Putamen |  | – |  | -26 | 8 | -8 | 5.79 |
| Frontal Sup. |  | 9 |  | -24 | 24 | 40 | 5.71 |
| Caudate |  | – |  | -14 | 14 | 12 | 5.44 |
| Frontal Inf. (pars triangularis) |  | 45 |  | -52 | 28 | 2 | 4.49 |
| Angular Gyr. | R | 39 |  | 62 | -54 | 34 | 6.97 |
| Putamen |  | – |  | 28 | 6 | -2 | 5.69 |
| Temporal Sup. |  | 22 |  | 56 | -30 | -6 | 5.07 |
| Caudate |  | – |  | 14 | 12 | 14 | 4.85 |
| **Cerebellum Crus 2** | **L** | – | **373** | **-20** | **-84** | **-36** | **4.38** |
| Cerebellum Crus 2 |  | – |  | -38 | -74 | -40 | 4.04 |
| **Hippocampus** | **R** | – | **104** | **30** | **-30** | **-12** | **3.98** |
| **Lat. Orbital Gyr.** | **R** | **47** | **160** | **36** | **34** | **-12** | **3.71** |
| Lat. Orbital Gyr. |  | 47 |  | 46 | 36 | -8 | 3.51 |
| **Precentral** | **L** | **44** | **71** | **-52** | **-4** | **16** | **3.58** |
| ***Short > Long context*** |  |  |  |  |  |  |  |
| **Parietal Sup.** | **R** | **7** | **25370** | **22** | **-56** | **56** | **Inf** |
| Postcentral |  | 3 |  | 48 | -28 | 54 | Inf |
| Occipital Inf. |  | 19 |  | 46 | -70 | -4 | 7.79 |
| Temporal Inf. |  | 37 |  | 46 | -60 | -4 | 7.65 |
| Parietal Sup. | L | 7 |  | -20 | -62 | 58 | Inf |
| Postcentral |  | 2 |  | -42 | -32 | 46 | Inf |
| Occipital Inf. |  | 19 |  | -46 | -72 | 0 | 7.57 |
| **Cerebellum vermal VI-VII** | **R** | **–** | **780** | **4** | **-66** | **-32** | **5.77** |
| Cerebellum lobule VIII |  | – |  | 16 | -68 | -50 | 5.04 |
| Cerebellum lobule VIII | L | – |  | -14 | -66 | -48 | 4.60 |
| **Precentral** | **L** | **44** | **337** | **-50** | **6** | **30** | **5.52** |
| **SMA** | **L** | **6** | **88** | **-6** | **-16** | **50** | **4.35** |
| **Precentral** | **R** | **44** | **103** | **50** | **8** | **26** | **4.22** |
| ﻿*Whole-brain activation cluster sizes (k), MNI coordinates (x, y, z), and Z-scores for the main effect of CONTEXT (long > short context and short > long context) (p_voxel_ < .001; correction for multiple comparisons to p < .05 was obtained using a voxel cluster extent threshold procedure which led to minimum cluster extent threshold of 46 re-sampled voxels). BA: Brodmann area, Hem.: hemisphere. Lat.: Lateral, Gyr.: Gyrus, Sup.: Superior, SMA: supplementary motor area.* | | | | | | | |
