# Supplementary material for: Lateral prefrontal cortex is a hub for music production from structural rules to movements
Source: Cereb Cortex. 2021 Dec 30;32(18):3878–95. doi: 10.1093/cercor/bhab454 (PMC9476625; doi:10.1093/cercor/bhab454)
Supplement: TableS2_accepted_211109_bhab454 [file tables2_accepted_211109_bhab454.docx]

| **Table S2. Full factorial analysis of trials from the ‘baseline’ and ‘motor’ blocks (Model 2).** | | | | | | | |
| --- | --- | --- | --- | --- | --- | --- | --- |
| Gyrus or region | Hem | BA | k | x | y | z | Z-value |
| ***Irregular > regular chord*** |  |  |  |  |  |  |  |
| **Parietal Sup.** | **L** | **8** | **3644** | **-22** | **-72** | **50** | **5.23** |
| Supramarginal Gyr. |  | 40 |  | -42 | -34 | 40 | 5.04 |
| Occipital Mid. |  | 19 |  | -32 | -82 | 28 | 4.42 |
| **Temporal Inf.** | **L** | **37** | **417** | **-46** | **-54** | **-14** | **4.97** |
| Occipital Inf. |  | 37/19 |  | -42 | -64 | -8 | 3.96 |
| **Frontal Mid.** | **L** | **8** | **1170** | **-24** | **-2** | **52** | **4.86** |
| Frontal Sup. |  | 6 |  | -20 | 0 | 52 | 4.62 |
| Precentral |  | 6 |  | -26 | -8 | 50 | 4.45 |
| **Frontal Inf. Op./Precentral** | **L** | **6** | **848** | **-46** | **6** | **30** | **4.54** |
| Frontal Inf. (pars Triangularis) |  | 45/44 |  | -54 | 26 | 16 | 3.72 |
| **Postcentral** | **R** | **3** | **317** | **44** | **-26** | **38** | **4.36** |
| Supramarginal Gyr. |  | 40 |  | 36 | -32 | 44 | 4.05 |
| **Frontal Sup.­­** | **R** | **8** | **293** | **22** | **-6** | **44** | **4.34** |
| “ |  | 6 |  | 24 | -10 | 52 | 3.92 |
| **Parietal Sup.** | **R** | **7** | **255** | **28** | **-76** | **52** | **3.92** |
| “ |  | 5 |  | 20 | -56 | 58 | 3.81 |
| ***Structure* × *Movement*** |  |  |  |  |  |  |  |
| **Postcentral** | **R** | **3** | **91** | **38** | **-36** | **66** | **3.75** |
| ﻿*Whole-brain activation cluster sizes (k), MNI coordinates (x, y, z), and Z-scores for the main effect of STRUCTURE (irregular > regular chord) and the STRUCTURE × MOVEMENT interaction (p_voxel_ < .001; correction for multiple comparisons to p <.05 was obtained using a voxel cluster extent threshold procedure which led to minimum cluster extent threshold of 46 re-sampled voxels). BA: Brodmann area, Hem.: hemisphere. Inf.: Inferior, Mid.: Middle, Sup.: Superior, Gyr.: Gyrus, Op.: pars opercularis.* | | | | | | | |
